# Neurobiological model of the persistence of anorexia nervosa

**DOI:** 10.1186/s40337-016-0106-2

**Published:** 2016-05-18

**Authors:** Joanna E. Steinglass, B. Timothy Walsh

**Affiliations:** 1051 Riverside Drive, Unit 98, New York, NY 10032 USA

**Keywords:** Anorexia nervosa, Cognitive neuroscience, Habit systems, Reward systems, Eating behavior

## Abstract

Anorexia Nervosa (AN) is characterized by the maintenance of an undernourished, or starved, state. Persistent restrictive eating, or the recurrent intake of a diet that is inadequate to sustain a healthy weight, is the central behavior maintaining AN. To understand this disturbance, we need to understand the neural mechanisms that allow or promote the persistent choice of inadequate caloric intake. While a range of neural disturbances have been reported in AN, abnormalities in systems relevant to reward processing and the development of habit systems have been consistently described in both structural and functional neuroimaging studies. Most recently, brain and behavior have been directly examined by investigating the neural underpinnings of restrictive food choice. These recent data suggest that, among individuals with AN, dorsal frontostriatal circuits play a greater role in guiding decisions regarding what to eat than among healthy individuals. This line of research attempts to leverage advances in the field of cognitive neuroscience to further our understanding of persistent maladaptive choices of individuals with AN, in the hope that such advances will help in the development of novel treatments for this potentially fatal disorder.

## Background

Anorexia Nervosa (AN) is characterized by the maintenance of an undernourished, or starved, state. Affecting approximately 1 % of women [1] across all socioeconomic classes [2], AN is characterized by severe restriction of food intake resulting in an inappropriately low body weight, fear and anxiety about weight gain, and preoccupation with body shape and weight [3]. The recurrent intake of a diet that is inadequate to sustain a healthy weight is the central behavior maintaining this condition. To understand this disturbance, it is useful to consider the neural mechanisms that promote the persistent choice of inadequate caloric intake, a behavior that clearly becomes maladaptive and self-destructive. Here, we review a cognitive neuroscience model of AN that focuses on the persistence of maladaptive behavior.

AN commonly begins during adolescence, with a peak age at first presentation between 14 and 18 years [4, 5]. Unlike many psychiatric illnesses, in which early onset signifies a more severe course of illness, adolescents with AN have a better prognosis than adults: studies of adolescents receiving treatment for AN show that, at 1 year of follow-up, approximately 75 % are in partial or full remission [6–8]. However, when the disease progresses into adulthood, the treatment outlook is disheartening [8, 9]. No pharmacotherapies have proven effective, psychosocial interventions are often inadequate, and relapse rates are high – up to 50 % of adult patients require rehospitalization within a year of discharge [10, 11]. Mortality among young women with AN is at least six times that expected for their age [12], the highest of any psychiatric disorder, and the likelihood of death increases with duration of illness [13]. A better understanding of the neural underpinnings of AN may help to develop novel treatments and improve outcomes for these severely ill individuals.

## Review

### The importance of eating behavior in AN

Eating is a multifaceted behavior, affected by multiple social, psychological and biological factors. The neurobiological value of food as a primary reward has been long noted [14–16], and much is understood regarding appetitive and inhibitory control around normal eating [17–19]. Yet, models of the neural mechanisms of normal eating have not contributed greatly to the understanding of eating disorders – perhaps because these models, largely based on studies of rodents, do not clarify the influences that promote maladaptive eating behavior among humans with eating disorders [19].

The aberrations in eating behavior that occur among individuals with eating disorders have been usefully examined via objective assessment in eating laboratories. Most such studies have compared the eating of individuals without eating disorders to that of individuals with binge eating, such as occurs in bulimia nervosa (BN) and binge eating disorder (BED). Laboratory studies have documented that individuals with BN and with BED consume significantly more calories than controls when asked to binge, and that some satiety cues are abnormal. These studies have also documented that, when not binge eating, individuals with BN tend to consume fewer calories than healthy volunteers [20, 21]. These studies laid a foundation for using the laboratory setting to examine abnormally reduced food intake such as occurs in AN.

Restrictive caloric intake in AN has been objectively assessed [22, 23]. One of the first observational studies of intake among individuals with AN demonstrated lower caloric intake compared with healthy controls (HC) (1289 ± 150 vs 2220 ± 108 kcal, *p* < 0.05) as well as restricted fat intake (17.6 ± 2.3 % vs 28.4 ± 1.3 % of total calories consumed, *p* < 0.05) [24]. Additional studies of eating behavior have demonstrated significantly restricted dietary intake among individuals with AN, not only when acutely ill but also immediately after weight restoration. In a single item meal study using an unfamiliar food, patients with AN ate significantly less than HC (104 ± 102 vs 490 ± 188 g, *p* < 0.01) and showed no significant improvement in intake even after they had gained to a normal weight (178 ± 203 g, *p* = 0.85) [25]. In another study, hospitalized patients with AN participated in a Multi-item Meal (www.phenxtoolkit.org [26]) in the laboratory before and after weight restoration treatment, and were compared with HC. Participants were brought to the eating laboratory where they were presented with an array of foods. Individuals with AN ate significantly fewer calories compared with HC, both at admission, when they were severely underweight (364 ± 208 vs. 775 ± 228 kcal, *p* = 0.001), and after full weight restoration (516 ± 273 vs. 758 ± 346 kcal, *p* = 0.03). Furthermore, individuals with AN were particularly avoidant of calories from fat at admission (18 ± 10%vs. 38 ± 7 % of total calories consumed, *p* = 0.001) and after weight restoration (23 ± 9 % vs. 38 ± 18 %, *p* = 0.004) [27]. Assessment of dietary histories of outpatients has also documented restricted intake [28] and specifically avoidance of fat among individuals with AN (15.7 ± 1.7 % vs. 24.8 ± 1.3 % of total calories consumed, *p* < 0.05) [24]. Critically, restrictive eating patterns including reduced caloric intake, limited diet variety and the consumption of foods with low energy density are related to poor outcome over time [29, 30].

“Dieting” is the colloquial term for restricting caloric intake, or, attempting to restrict caloric intake below the amount required to maintain weight. Self-reported dieting is almost universal among adolescent girls, and occurs in up to 75 % of healthy adult populations as well [31]. In the majority of individuals, dieting is ineffective in altering long term weight [32, 33]. For as many as 38 % of dieters, the behavior becomes frankly pathological, termed “disordered eating” [34, 35], and 25 % of those individuals develop eating disorders [36]. Dieting is a signature behavior across eating disorders [37], where persistent efforts to restrict intake are accompanied by disturbed psychological functioning. AN is persistent dieting at its most extreme. Consideration of the importance of dieting in eating disorders is not new – some approaches to probing the neurobiology of AN have usefully incorporated hypotheses about the relationship between neurobiology and dieting, possibly as a “self-medication” for anxiety [38, 39]. Yet, the neural mechanisms of persistent, maladaptive caloric restriction – a signature behavior of several eating disorders - have not been rigorously examined.

Eating is complex and multi-determined [40, 41]. However, the findings described above demonstrate that the salient behavioral disturbance of individuals with AN is the selection of low-calorie foods in a remarkably stereotyped fashion that promotes persistence of illness. A logical and important next step is to ask what drives this behavior neurobiologically.

### Cognitive neuroscience of anorexia nervosa

Cognitive neuroscience aims to illuminate the neural basis of human behavior, and to link advances in psychology with biological science. This approach has great potential as we aim to understand the neural mechanisms of the salient maladaptive behaviors in AN. Some proposed models have used a “bottom-up” approach, in which basic processes are studied in order to evaluate aberrancies in brain functioning. For example, study of cognitive domains (e.g., set shifting [42–44]), showed neuropsychological abnormalities; individuals with AN have more difficulty shifting between cognitive tasks, perhaps related to a tendency toward psychological rigidity. However, cognitive findings are not entirely consistent across studies, and these broad executive functioning deficits do not identify specific underlying neural targets [45, 46]. A similar “bottom-up” approach examined basic processes such as taste and yielded evidence of differences in neural activation related to probes of images and actual sensory processing [38, 47]. But, these behavioral and neural abnormalities have not been directly linked to eating disorder behavior. Nonetheless, these studies, and others reviewed below, have consistently shown abnormalities in reward systems and in frontostriatal systems and have paved the way for our current, “top-down,” approach that probes the neural activity directly related to disturbances in eating behavior.

#### The neurobiology of reward in AN

Mesolimbic neural systems of reward processing encompass the midbrain/ventral tegmental area, ventral striatum (including nucleus accumbens, NAcc), and orbitofrontal cortex (OFC). Several studies have examined neural correlates of reward processing among individuals with AN using structural and functional MRI, as well as PET. Structural MRI studies have shown volumetric abnormalities among individuals with AN within reward-sensitive regions, including the OFC, though the directionality of these abnormalities (increased vs decreased volumes) has been inconsistent [48, 49]. Task-based fMRI studies have examined responses to monetary and taste rewards in AN. When asked to respond to monetary stimuli, individuals with AN showed abnormalities in striatal and prefrontal cortex activity [50, 51]. Abnormal task-related responses to images of food and receipt of sweet vs. non-sweet liquids have also been shown with abnormally increased activity within the OFC and the NAcc [52–54]. One PET study indicated increased dopamine receptor density in the NAcc of 10 women recovered from AN [55]. However, Broft et al. found no differences in striatal dopamine receptor binding potential in AN nor any impact of weight gain [56]. Broadly, across a range of imaging techniques and study designs, studies suggest abnormalities within reward circuitry in AN. Early data also indicate abnormalities in the organization of this neural circuit, with heightened connectivity based on resting fMRI and diffusion tractography MRI between the NAcc and the OFC among adolescents and young adults with AN [57].

The functioning of reward systems in AN is likely impacted by both food restriction and adolescence itself. Studies in rodents suggest that food restriction sensitizes reward circuits [58]. For example, following 1-week of food restriction, rats showed increases in dopamine release in response to cocaine and amphetamines [59]. Food restriction in rodents and non-human primates resulted in subsequent increases in drug self-administration [58, 60]. Studies of reward systems in adolescence have also shown increased salience of rewards. For example, an fMRI study compared activation of the NAcc and OFC in children, adolescents, and adults during a task that paired stimulus selection with varying amounts of monetary reward. Relative to the adults and children, the teens showed the strongest blood-oxygenation level dependent (BOLD) signal response within the NAcc to the monetary rewards [61]. Similarly, in a Go/No-Go Task that induces a decrease in impulse control with rewarding relative to neutral stimuli, adolescents showed the largest decrement in impulse control and this behavior was associated with increased NAcc activity [62]. Since AN is defined by food restriction and usually begins during adolescence, these studies underscore the potential relevance of the organization and function of reward systems in AN. Indeed, several reward-centered models of the pathophysiology of AN have emerged [63–67]. While these models differ somewhat in their focus, they overlap in suggesting that AN is associated with abnormalities in the mesolimbic reward systems.

#### The neurobiology of frontostriatal systems in AN

Habit formation is the process by which a behavior associated with receipt of a reward, if repeated frequently (“practiced”), becomes almost automatic and far less dependent on the receipt of the reward [68]. As behavior shifts from goal-directed to habitual, there is an accompanying shift in the neural systems supporting behavior [69–71]. Human and animal research indicate that once the behavior achieves the characteristics of habit (i.e., becomes outcome independent), it is under the control of neural systems that include the dorsal striatum (basal ganglia, caudate and putamen) and associated dorsolateral frontal cortex. These circuits are thought to be of particular interest in persistent, maladaptive behaviors seen across psychiatric illnesses [72].

A range of disturbances in dorsal frontostriatal systems has been described in AN. PET studies have shown hypermetabolic abnormalities in the caudate among patients with AN, as compared with healthy controls (HC) and with individuals with BN [73–76]. A PET dopamine binding study also reported that dopamine binding potential in the dorsal caudate correlated with harm avoidance, a trait commonly found in individuals with AN [55]. Structural studies, including a meta-analysis, have shown decreased volume in the caudate among individuals with AN [48]. One fMRI study of a monetary guessing game found increased neural activity in the caudate among recovered AN [50]. A study using food pictures for symptom provocation found greater caudate activation among individuals who had recovered from AN as compared with HC [77]. These data have yielded several models proposing frontostriatal dysfunction in AN [65, 78–82].

### A habit centered model

We have recently suggested that persistent dieting in AN has the behavioral characteristics of “habit” [68]: restrictive intake in AN is learned, not innate, occurs repeatedly, and, once learned, is elicited by specific stimuli for the individual [78]. As these behaviors are learned through reinforcement, the mesolimbic reward system is highly relevant for understanding the development and persistence of AN. Habitual behaviors are defined as being relatively insensitive to the receipt of a desirable outcome –a person continues the behavior even if their desire for the outcome has changed [69]. Most importantly, once the behavior is established, it takes great effort from the individual to change the behavior. Individuals with AN develop – i.e., learn - dieting behavior, typically during adolescence. The dieting behavior is reinforced for a number of reasons, many specific to the affected individual (e.g., the pleasure of achieving weight loss; the receipt of compliments; a sense of satisfaction for having achieved a challenging goal, a heightened sense of self-control). As noted above, adolescence is a stage of life when reward salience is unusually high, which may also contribute to the development and perpetuation of behaviors in young people [61, 62]. Among individuals with AN, the effects of starvation on the brain likely also contribute to altered decision-making. The dieting behavior is repeated, and with repetition becomes more automatic, such that ultimately the behavioral routines are elicited by cues (e.g., the onset of a meal leads to initiation of a series of ritualized behaviors that collectively serve to minimize intake).

A key prediction of the habit model of AN is that restrictive intake in AN is associated with activity in the dorsal striatum. We recently tested this hypothesis in a group of older adolescents and adults with AN, using a Food Choice Task performed during fMRI scanning [83]. In this task [84], participants are asked to make a series of choices between a food that they themselves have rated as “Neutral” on both healthiness and tastiness, and a series of 75 other food items. Participants are aware that after the task they will receive one of their selections to eat as a snack, so their choices have real implications. As expected (and as previously shown [84]), the participants with AN were substantially less likely to choose the high fat food items (F_1,40_ = 32.2, *p* < 0.0001).We also found that caloric intake in the laboratory meal the following day was significantly associated with proportion of high fat items selected in the task (*r* = 0.61, *p* < 0.01), indicating that this assessment captures the restrictive intake so characteristic of AN.

In order to examine the neural correlates of the behavior, we measured the correlation between BOLD signal and each participant’s choices by entering individual choice ratings in a parametric analysis to determine the link between behavior and BOLD activity. Whole brain analyses in controls and individuals with AN during food choice showed no significant differences between groups in the mesolimbic reward regions (even at a lenient threshold), but significantly different patterns of activation in the dorsal striatum. Region of interest (ROI) analyses focused on the dorsal striatum, as per the *a priori* hypothesis, and, as hypothesized, in AN, food choices were strongly associated with dorsal striatum activity, a pattern that was not found in the control group (*p* < 0.05).

This study also yielded intriguing behavioral findings that demonstrate the complex relationship between “self-control” and “habit” in maladaptive eating behavior. Individual ratings of healthiness and tastiness of each food item were used to identify trials in which “self-control” would need to be engaged to make the “correct” (i.e., healthy) choice; that is, to choose foods that were Healthy even when they were rated by the participant as Not Tasty, or, conversely, to *not* choose foods rated Not Healthy but Tasty [85]. Compared to controls, individuals with AN tended to rate Not Healthy foods as Not Tasty, leading to their having fewer choices in which “self-control” was needed (t_40_ = 3.10, *p* = 0.004). Even so, individuals with AN made the self-controlled choice significantly more often when the opportunity arose (t_40_ = −4.89, *p* = 0.00002).

## Conclusions

The origins of AN are complex. Our current understanding is that innate factors, including genetic ones, contribute significant risk but are not determinative. Acting on such underlying vulnerabilities, psychosocial and environmental factors, typically combined with the stresses of adolescence, trigger a commitment to calorie restriction and weight loss that initially appears no different from that experienced by a majority of adolescent girls in developed countries. In those who develop AN, however, food restriction becomes all-consuming, relentless, and remarkably stereotyped. Among the substantial number who develop chronic illness, dietary practices are deeply entrenched and difficult to alter, underlying the persistence of the disorder.

The stereotyped nature of the behaviors characteristic of AN and their persistence suggest that affected individuals share similar disturbances in neural circuits related to restrictive food intake. The field of cognitive neuroscience aims to understand how the brain governs mental processes and ultimately behavior. We have adopted a “top-down” approach based in cognitive neuroscience to attempt to elucidate the core behavioral disturbances of AN. In this model, we propose that some aspect of dieting behavior is initially rewarding, but that this behavior persists in individuals with AN as maladaptive behavior because it is ultimately mediated by neural circuits linked to habit formation.

We and others have found that the characteristic patterns of food restriction can be readily assessed by objective measurement of eating behavior. By adapting techniques utilized by cognitive neuroscientists to examine the neural basis for self-control in avoiding “junk food,” we have developed a Food Choice Task using images of foods that accurately captures the restriction of caloric intake of AN and that is predictive of actual food intake. Conducting this task during fMRI, we have found that the neural circuits engaged by individuals with AN during food choice differ significantly from those engaged by healthy controls, and are circuits associated with habit formation.

We emphasize that this line of work has only just begun, and that many questions are unanswered. The recently acquired fMRI data addressed the hypothesis that persistent maladaptive behavior in AN is mediated by dorsal frontostriatal circuits that underlie habit behavior. Yet, this was not a definitive test of whether restrictive intake is a “habit.” Additionally, it is not known if the neural circuits engaged by successful dieters who do not develop AN differ from those of individuals with the disorder. It is not known how the neural circuits related to food choice change as AN becomes chronic nor how they change with treatment. There is compelling evidence that psychological and emotional factors impact food restriction in AN, and it is not clear how such factors affect the neural circuits associated with food choice. However, it is our hope that the integration of the perspective of cognitive neuroscience into the study of AN will be a source of new lines of thinking and of probing this enigmatic disorder.
